# 701. Predicting hospital-associated *Clostridioides difficile* infection risk in hospitalized patients using routinely-collected electronic health record data

**DOI:** 10.1093/ofid/ofad500.763

**Published:** 2023-11-27

**Authors:** Michael J Ray, Luke Strnad, Jon P Furuno, Eric Lofgren, Jeffrey S Gerber, Jessina C McGregor

**Affiliations:** OSU/OHSU College of Pharmacy, Portland, Oregon; Oregon Health and Science University, Portland, Oregon; Oregon State University, Portland, Oregon; Washington State University, Pullman, WA; Children's Hospital of Philadelphia, Philadelphia, Pennsylvania; Oregon State University, Portland, Oregon

## Abstract

**Background:**

Antibiotic therapy is a known risk factor for *Clostridioides difficile* infection (CDI), though the risk varies by agent. The antibiotic spectrum index (ASI) captures information on spectrum of antibiotic activity and days of therapy (DOT). We evaluated ASI and other routinely collected clinical data as predictors of hospital-associated (HA) CDI.

**Methods:**

We developed and tested a model to predict HA-CDI in a cohort of adult inpatient encounters at a 576-bed academic medical center in Portland, OR. We excluded patients with recurrent or community-acquired CDI. We used encounter data from 2/2018 – 2/2020 to train and validate our model. Using a randomly-selected two-thirds of our data, we trained our logistic regression model using a stepwise variable selection process. We used the remaining one-third of our data to validate our model. Finally, we applied the model to the 3/2020 – 3/2021 period to test the model’s ability to predict HA-CDI. We generated receiver operating characteristic (ROC) curves and calculated area under the curve (AUC) to evaluate model discrimination. Finally, we compared observed and predicted HA-CDI counts by decile using the Hosmer-Lemeshow test.

**Results:**

The training sample included 84 HA-CDI cases among 23622 encounters. The final model included ASI per DOT, time at-risk (days), patient age, number of hospitalized days in the 8 weeks prior, and inpatient receipt of a proton pump inhibitor or H2 receptor antagonist. When applied to the validation set (44 cases/11807 encounters), the model achieved an AUC of 0.79 (95% Confidence Interval: 0.71, 0.86) When applied to the test period (35 cases/12341 encounters), the AUC was 0.73 (0.66, 0.81). According to the Hosmer-Lemeshow test, our model fit the data reasonably well (p=0.2; Figure 1). Using ASI in the model achieved a significantly better prediction than DOT (Figure 2).

**Figure:**
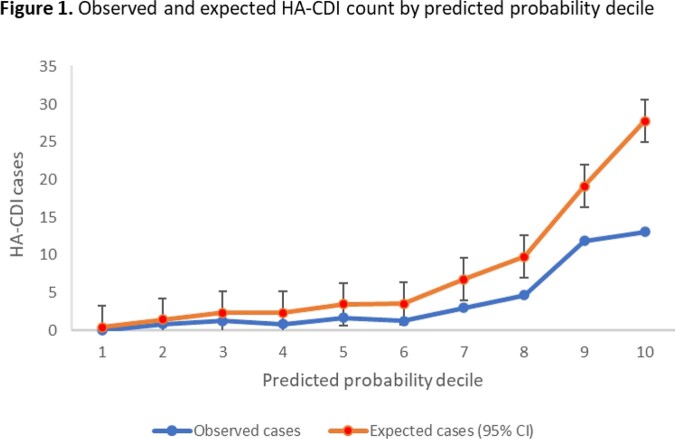

**Figure:**
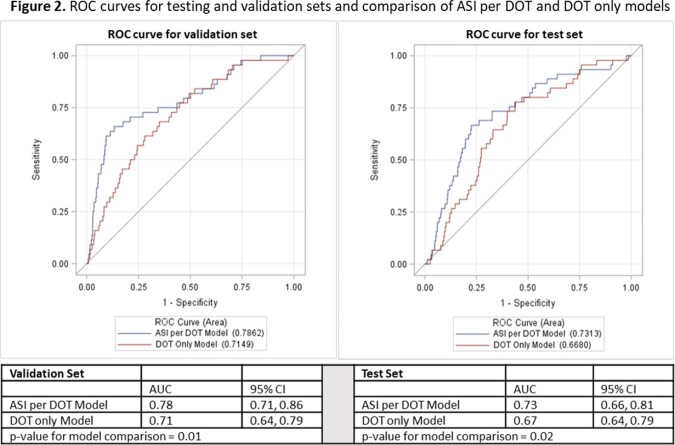

**Conclusion:**

Using a combination of ASI and data that is routinely-collected for clinical practice, we were able to reasonably predict HA-CDI in our hospital using a small number of variables easy-to-obtain variables. The antibiotic spectrum index could be a valuable tool in predicting individual-level HA-CDI risk, though further work is required to test and implement a CDI risk score in a prospective environment.

**Disclosures:**

**All Authors**: No reported disclosures

